# Effector-dependent stochastic reference frame transformations alter decision-making

**DOI:** 10.1167/jov.22.8.1

**Published:** 2022-07-11

**Authors:** T. Scott Murdison, Dominic I. Standage, Philippe Lefèvre, Gunnar Blohm

**Affiliations:** 1 Centre for Neuroscience Studies, Queen's University, Kingston, Ontario, Canada; 2 Canadian Action and Perception Network (CAPnet), Toronto, Ontario, Canada; 3 Association for Canadian Neuroinformatics and Computational Neuroscience (CNCN), Kingston, Ontario, Canada; 4 Centre for Neuroscience Studies, Queen's University, Kingston, Ontario, Canada; 5 Canadian Action and Perception Network (CAPnet), Toronto, Ontario, Canada; 6 Association for Canadian Neuroinformatics and Computational Neuroscience (CNCN), Kingston, Ontario, Canada; 7 School of Psychology, University of Birmingham, UK; 8 ICTEAM Institute and Institute of Neuroscience (IoNS), Université catholique de Louvain, Louvain-La-Neuve, Belgium; 9 Centre for Neuroscience Studies, Queen's University, Kingston, Ontario, Canada; 10 Canadian Action and Perception Network (CAPnet), Toronto, Ontario, Canada; 11 Association for Canadian Neuroinformatics and Computational Neuroscience (CNCN), Kingston, Ontario, Canada

**Keywords:** decision-making, reference frames, visuomotor, head roll, motion perception

## Abstract

Psychophysical, motor control, and modeling studies have revealed that sensorimotor reference frame transformations (RFTs) add variability to transformed signals. For perceptual decision-making, this phenomenon could decrease the fidelity of a decision signal's representation or alternatively improve its processing through stochastic facilitation. We investigated these two hypotheses under various sensorimotor RFT constraints. Participants performed a time-limited, forced-choice motion discrimination task under eight combinations of head roll and/or stimulus rotation while responding either with a saccade or button press. This paradigm, together with the use of a decision model, allowed us to parameterize and correlate perceptual decision behavior with eye-, head-, and shoulder-centered sensory and motor reference frames. Misalignments between sensory and motor reference frames produced systematic changes in reaction time and response accuracy. For some conditions, these changes were consistent with a degradation of motion evidence commensurate with a decrease in stimulus strength in our model framework. Differences in participant performance were explained by a continuum of eye–head–shoulder representations of accumulated motion evidence, with an eye-centered bias during saccades and a shoulder-centered bias during button presses. In addition, we observed evidence for stochastic facilitation during head-rolled conditions (i.e., head roll resulted in faster, more accurate decisions in oblique motion for a given stimulus–response misalignment). We show that perceptual decision-making and stochastic RFTs are inseparable within the present context. We show that by simply rolling one's head, perceptual decision-making is altered in a way that is predicted by stochastic RFTs.

## Introduction

We typically maintain upright head and eye orientations with respect to the horizon (Pozzo et al., 1990; Dunbar et al., 2004, 2008), despite potentially increased energy expenditure. For example, during hunting (Land, 2014), flight (Altshuler et al., 2015), or motorcycle racing, it would be more energy efficient to align the head with the inertial vector. Minimizing vertical disparity has been suggested as one reason for this behavior (Misslisch et al., 2001; Schreiber et al., 2001).

A complementary reason could be that reference frame transformations (RFTs) are stochastic (Alikhanian et al., 2015), that is, RFTs depend on internal, noisy (stochastic) estimates of transformation parameters, such as rotation angles. Signal-dependent noise in the RFT parameters then leads to added variability in transformed signals. The effect of such stochastic RFTs is apparent in both perception (Schlicht & Schrater, 2007; Burns et al., 2011) and motor planning (Sober & Sabes, 2003, 2005; McGuire & Sabes, 2009; Burns & Blohm, 2010; Abedi Khoozani & Blohm, 2018). If the encoding of visual motion evidence is similarly degraded by stochastic transformations, then maintaining specific head orientations while making decisions about its velocity could be optimal for the signal's preservation, despite requiring energy expenditure (Umberger et al., 2003).

**Figure 1.**
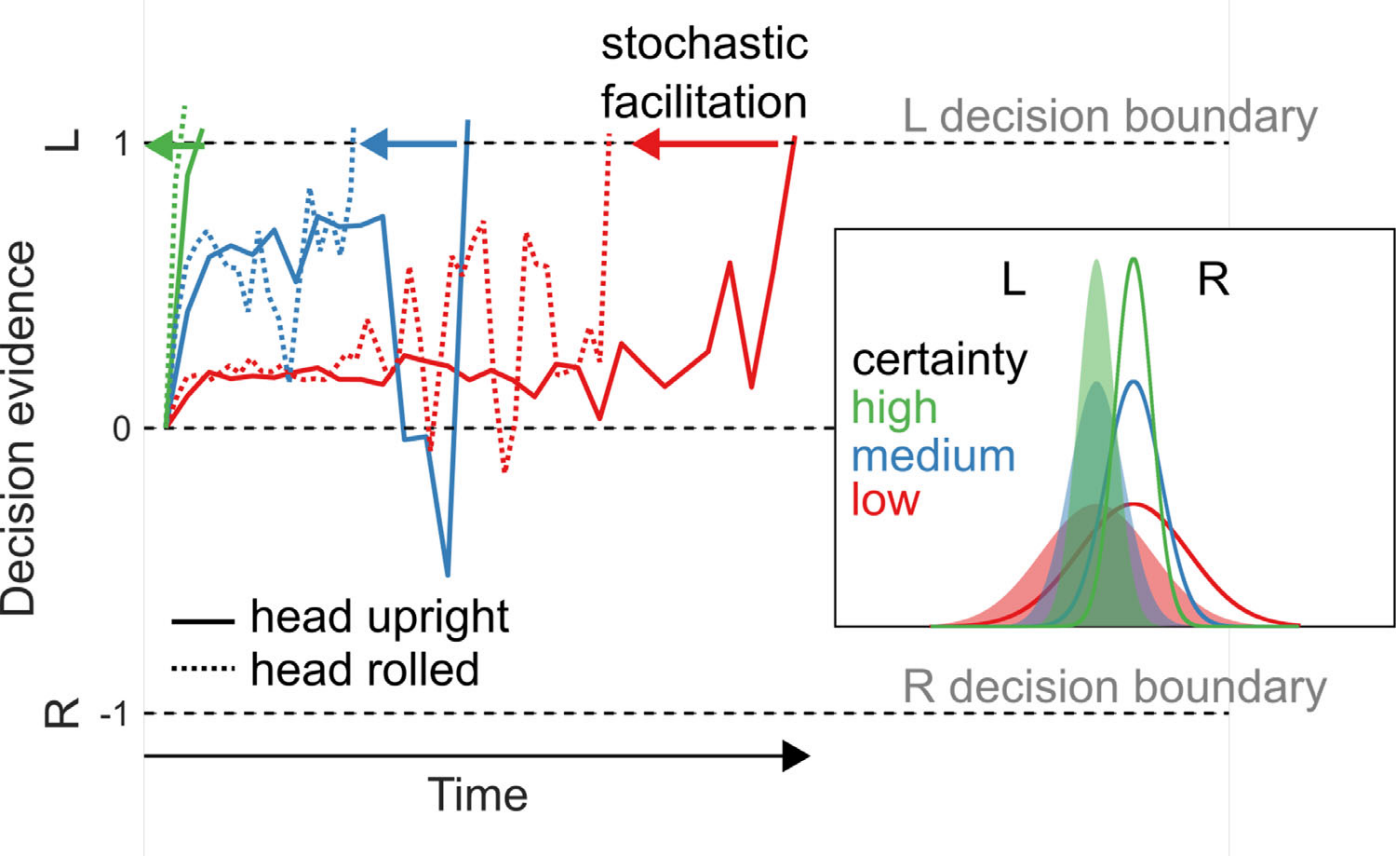
Potential roles of noise in perceptual decision-making. Six separate perceptual decision processes (three different evidential certainties with head upright/rolled) are simulated within a drift–diffusion framework for leftward target motion (see shaded curves in inset). One possible role for RFT noise is in the degradation of motion evidence certainty (modeled by Gaussian distributions), which can be seen in the inset. Another possible role for RFT noise is in stochastic facilitation of the decision dynamics (dotted lines). Leftward color-matched arrows represent theoretical influence of stochastic facilitation on response times. Evidence accumulation in this illustrative model is represented by the summed log ratios for random draws from each distribution, biased in the leftward direction and with uniform noise added to the signal.

Bounded accumulator models account for a wealth of behavioral data from perceptual decision tasks under the premise that noisy evidence for the alternatives is accumulated until it reaches a criterion bound (Smith & Ratcliff, 2004; Bogacz et al., 2006). Under this framework, stochastic RFTs could influence choice behavior in predictable ways. One possibility is that RFTs can degrade the encoding of evidence by lowering its signal-to-noise ratio. An example of such evidence degradation is shown for a simple theoretical decision process in Figure 1 (see inset containing Gaussian distributions). In this case, the decision-making performance should match the expectations of increasing task difficulty: increased reaction times (RTs) and decreased accuracy (percent correct). A complementary hypothesis (Standage, Wang, et al., 2014) is that different levels of noise result in different neural decision dynamics (Faisal et al., 2008), changing the balance between speed and accuracy. In this case, we would expect to observe faster and less accurate decisions by pushing decision circuitry into a regime with faster dynamics (and therefore less temporal integration). Direct evidence for either of these hypotheses is lacking (for review, see Standage, Blohm, et al., 2014).

A third possible role for noise in the perceptual decision process is stochastic facilitation. In this scenario, the presence of relevant, noisy endogenous signals (e.g., head and eye orientation afferents) improves information processing through an enhancement of neural signals. Stochastic facilitation has been shown to benefit many neural processes across different behavioral paradigms, animal models, and computational frameworks (for review, see McDonnell & Ward, 2011). RFTs may similarly provide a benefit to perceptual decision-making through faster, but no less accurate, decisions. A model illustrating stochastic facilitation's possible role in a theoretical drift–diffusion decision process for three levels of certainty for sensory evidence is shown in Figure 1.

**Figure 2.**
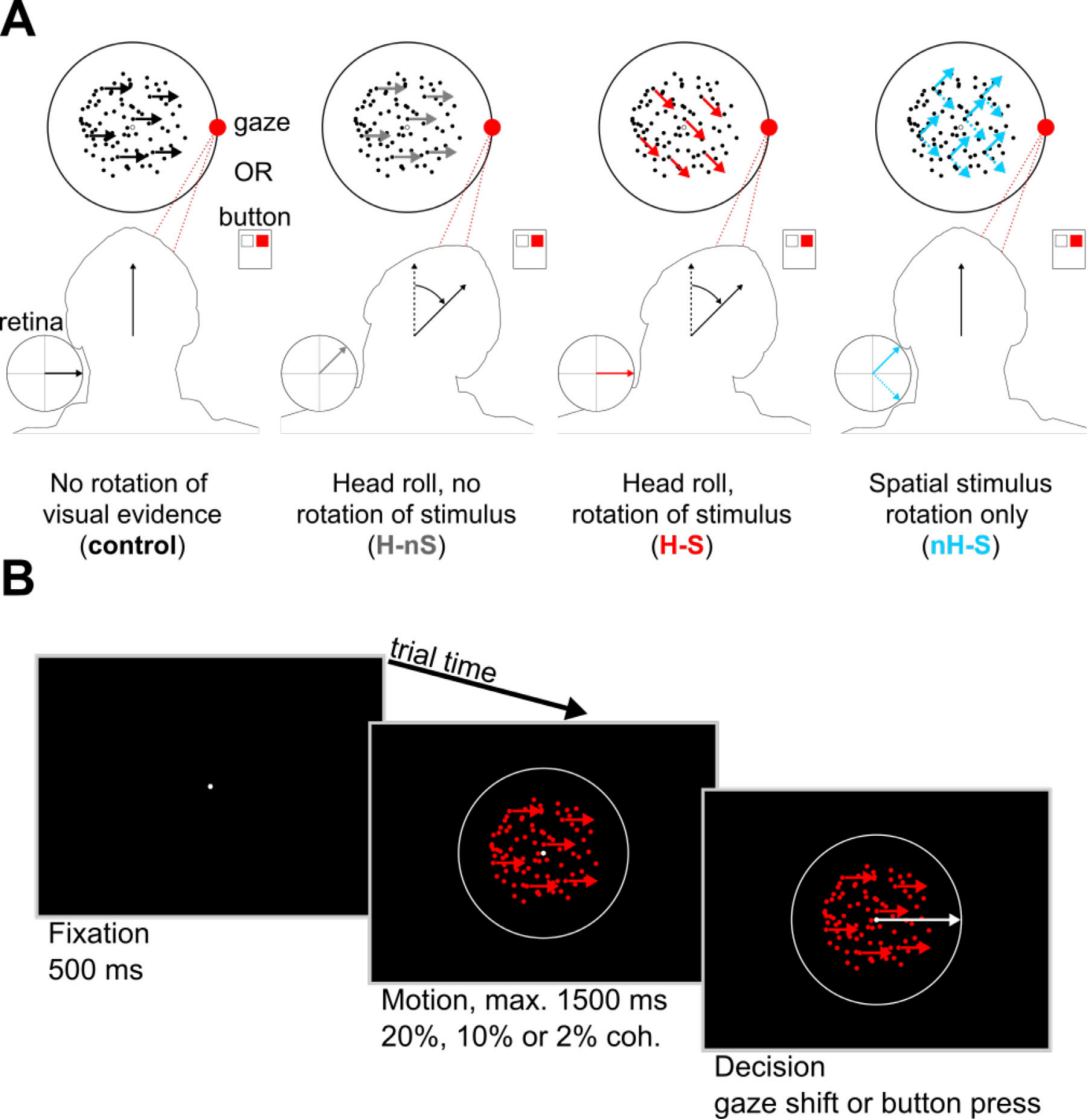
Task and paradigm. (**A**) Participants performed the task under one of eight conditions—four for each response type (saccade or button), organized in a block design. These were combinations of head and/or congruent screen rotations, giving rise to visual motion that was separable across eye, head, and shoulder (screen) reference frames. (**B**) Each trial consisted of a fixation (500 ms), motion (up to 1,500 ms), and decision epoch. Participants were instructed to determine the direction (left or right) of coherently moving dots randomly chosen at 20%, 10%, or 2% coherence and make their decision using either a horizontal saccade or a button press as quickly and accurately as possible.

The goal of this study was to determine the influence of stochastic RFTs on perceptual decision-making. To do so, participants were asked to perform a two-alternative, forced-choice (2AFC) random dot motion direction discrimination task either under nonrotated (control) or under head roll and/or rotated stimulus conditions (Figure 2). In a blocked design, they were also instructed to indicate their decision regarding the left or right direction of coherent visual motion with either a saccade or a button press. Because eye movements are executed in head-centered coordinates, and, when the arm is stationary, button presses occur in shoulder-centered coordinates, this paradigm allowed us to correlate decision performance effects with different visuomotor RFT requirements.

## Materials and methods

### Participants

Seven participants were recruited for the experiment after informed consent was obtained. Six of seven participants were naive as to the purpose of the experiment. Participants were between the ages of 22 and 32 years (five male), and all were right-handed. We also recruited five more participants for a second experiment with seven participants (two participants were in both experiments), representing an instance of our reaction time task with no deadline. Participants in this experiment were between the ages of 20 and 26 years (four male), and six of seven were right-handed. All participants had normal or corrected-to-normal vision and did not have any known neurological, oculomotor, or visual disorders. All procedures were approved by the Queen's University Ethics Committee in compliance with the Declaration of Helsinki.

### Experimental paradigm

To test how RFTs affect perceptual decisions, we developed an experimental paradigm with distinct conditions consisting of (1) rotations of the visual stimulus, (2) rotations of the head, and (3) changes to the response type (saccade or button press). These conditions allowed us to comprehensively investigate the influence of different RFTs on the decision process based on the coding frame of the motion evidence and transformation of that evidence into a reference frame appropriate for the motor response. These conditions are illustrated in Figure 2A.

We determined participants’ baseline decision-making performance using a control condition in which participants’ heads remained upright (0° roll) and the axis of coherent motion remained along the horizontal (0°) screen-centered axis. Thus, comparing our other experimental conditions to this one provided the effects directly resulting from adding new requirements to the transformation (Figure 2A, first column). For each response type, the rotational conditions were rolling the participants’ heads toward a shoulder, without rotation of the on-screen stimulus (head roll–no stimulus rotation, H-nS, Figure 2A, second column); head roll with 45° rotation of the on-screen stimulus (head roll–stimulus rotation, H-S, Figure 2A, third column); and 45° rotation of only the on-screen stimulus (nH-S, Figure 2A, fourth column).

### Apparatus

Participants sat in complete darkness 50 cm in front of a 36-cm × 27-cm Dell UltraScan P991 CRT monitor (Dell, Round Rock, TX, USA). Participants’ heads rested on a chinrest that allowed for head roll in the frontoparallel plane. With their heads in an upright position on the chinrest, the interocular midpoint was aligned to the frontoparallel fixation position on the screen. The visual stimulus was displayed on the screen (120 Hz refresh rate) using the ViSaGe Visual Stimulus Generator with VSG Toolbox for MATLAB (Cambridge Research Systems, Rochester, UK). Movements of both eyes were recorded at 400 Hz using a Chronos head-mounted 3D video eye tracker (Chronos Vision, Berlin, Germany) that was stabilized to the head using a bite bar. Although torsional eye movements were recorded by this system for some participants, these data were unfortunately inconsistent both within and between participants due to poor iris illumination and/or focus. For the purposes of our correlational analyses, we therefore assume a small (e.g., ∼10%) ocular counter-roll gain for the contribution of ocular torsion to the rotation of retinal input relative to head roll based on previous experimental findings (Blohm & Lefèvre, 2010; Murdison et al., 2013). Head movements were recorded at 400 Hz using an Optotrak Certus system (Northern Digital, Waterloo, Ontario, Canada) with three infrared diode markers placed on the Chronos helmet. For consistency in head orientation measurements across slight differences in camera positions, these helmet markers were calibrated with respect to an external orthonormal axis defined by a set of three orthogonal diodes located either on the wall behind the participant or on the side of the CRT monitor. Screen brightness and contrast settings were adjusted so that participants could not see the edges of the monitor screen in complete darkness, even after 0.5 hours of dark adaptation.

### Procedure

The visual stimulus consisted of a centered array of white circular dots (each 0.1° diameter) arranged in a circle (10° diameter), marking the boundary to which participants were instructed to make saccadic responses. At the center of this boundary, there was an aperture (5° diameter) inside of which we displayed the random dot motion stimulus. The central stimulus was composed of a white fixation point (0.1° diameter) positioned at the center and 200 red dots (each 0.1° diameter) with constant speeds of 4°/s. We chose to make all moving dots red to minimize CRT phosphor decay time (avoiding streaking across the screen). On each trial, we randomly selected a subset of the dots in motion (2%, 10%, or 20% of all dots) to move coherently in either the leftward or rightward direction. In the stimulus rotation conditions (H-S and nH-S), we rotated the on-screen motion axis by either 45° or –45°. In the H-S condition, this on-screen rotation of motion was congruent with the direction of head roll, such that the motion axis lay approximately along the interocular axis. In all saccadic trials, participants were instructed to make eye movements toward the on-screen 0° (rightward motion) or 180° (leftward motion) directions. The noncoherent dots had an average path length of 4 pixels, after which their direction was randomly determined on the interval from 0° to 360°. Participants were also informed of all block conditions (i.e., head roll, visual stimulus rotation) prior to the start of each block.

A sample trial progression is illustrated in Figure 2B. At the start of each trial, a fixation dot appeared in the center of the circular saccade boundary (fixation period, 500 ms). This fixation period was followed by the visual motion stimulus, displayed within the aperture in the center of the screen along with the fixation point (1,500 ms max). Participants were instructed to maintain fixation until they came to a decision about the direction of the coherent motion and were asked to do so as quickly and as accurately as possible. Depending on the response condition, they either made a saccade along the screen-centered horizontal (left or right) or pressed a button with their right hand's index or middle finger corresponding to the perceived horizontal component of motion (index for leftward motion, middle for rightward motion). For saccade response trials, participants were instructed to press any button after making a saccade, ending the trial. Importantly, we made the assumption that any preparatory activity related to this button press (which always followed the saccade response) did not impact eye movement–related decision processes due to significant elapsed time between the decision and the button press. For button press trials, the decision also ended the trial. Participants were not given feedback about whether their response was correct. There was an intertrial interval of 500 ms during which the screen was completely black.

Each participant performed four sessions, each consisting of seven 100-trial blocks for a total of 2,800 trials. All 14 conditions (left and right head rolls and stimulus rotations included) were counterbalanced across all participants using a reduced Latin squares method with an initially randomized list of all conditions (Shao & Wei, 1992). To counterbalance potential learning and fatigue effects, participants performed each condition twice: once in an initial sequence determined by the Latin squares method and a second time in the reverse sequence. Using this method, each condition was uniformly distributed across all blocks.

### Raw signal analysis

Three-dimensional (3D) head orientation was computed offline as the difference (using quaternion rotation based on Leclercq et al., 2013) between a reference upright position measured at the start of each experimental session and head position throughout the trials. Participants were instructed to begin the first block of each experimental session with an upright head position before responding to the verbal head roll instruction.

The eye-in-head orientation was extracted and calibrated, and saccades were detected using the same techniques as those used by previous work (Blohm & Lefèvre, 2010; Murdison et al., 2013). Briefly, the eye-in-head orientation was extracted after each session from the saved images of the eyes using Iris software (Chronos Vision). This was done using a 9-point grid of calibration dots (10° max eccentricity) with a central fixation point, while the head remained upright on the chinrest. Each participant was fitted with a customized bite bar to stabilize the Chronos helmet to the head. Eye-in-head orientation was low-pass filtered (autoregressive forward-backward filter, cutoff frequency = 50 Hz) and differentiated twice (weighted central difference algorithm, width = 5 ms). Saccades were detected using an acceleration threshold of 500°/s^2^, as previously done (Blohm & Lefèvre, 2010; Murdison et al., 2013). We defined the eye movement direction as the circular average of horizontal and vertical eye velocity components over the duration of the saccade. For each trial, the head roll measurement was obtained by taking the average head orientation from the motion stimulus onset until the decision time.

### Trial selection

For the main experiment, we recorded a total of 19,600 trials from seven participants (2,800 trials per participant from four sessions of seven 100-trial blocks each). Of those trials, we removed those that contained a head movement, blink, optokinetic nystagmus, or smooth pursuit movement after motion stimulus onset but prior to the decision. Finally, we removed trials on which participants had reaction latencies shorter than 200 ms (visuomotor processing delays) (Thorpe et al., 1996), as these trials likely corresponded to decisions made preemptively without the use of the visual motion evidence. From the extracted saccades and button presses, we determined trial-to-trial directional choices and computed cumulative RT distributions for each rotational condition. For saccades, left or right decisions were classified as saccades whose average direction (based on the entire movement) was within a conservative directional window around the screen-centered horizontal direction (0° or 180°) with a width of ± 75°. Trials with saccades with directions outside these windows were removed from the analysis. Also, trials for which the participant failed to respond before the end of the 1,500-ms response period were removed from analyses (14% of all trials). Together, these omitted trials comprised 22% of all trials, leaving 15,274 valid trials.

### Behavioral analysis

We quantified task performance using three main behavioral parameters capturing both speed and accuracy aspects of task performance. These parameters were RT (time elapsed between motion stimulus onset and response), percent error (number of valid incorrect trials divided by the total valid correct and incorrect trials multiplied by 100; conversely, percent correct = 100%-percent error), and reward rate (sum of the number of correct trials divided by the sum of all correct and incorrect reaction times). From these parameters, we computed the cumulative RT distributions for correct and incorrect trials, to which we fit a modified version of the linear approach to threshold with ergodic rate (LATER) model (Carpenter & Williams, 1995).

Because of the short 1,500-ms response window, some RT distributions were truncated, resulting in LATER-estimated RT distributions that were not necessarily representative of the data. To account for this issue, we fit both correct and incorrect trial RT distributions simultaneously using estimated percent correct as a free parameter that scaled each distribution relative to the other (correct representing percent correct or 100%-percent error at RT = ∞). We also performed all analyses with the empirical percent correct using just the trials within the 1,500-ms window and found results qualitatively similar to those based on the estimated percent correct. We performed the fits using a constrained nonlinear method that minimized the sum of squared residuals. These LATER model fits to the cumulative RT distributions revealed the estimated median reaction time with its µ parameter, the approximate slope of the distribution (representing the variability of the distribution) with its σ parameter, and the estimated percent correct, each of which we used in behavioral analyses.

To capture behavioral differences across conditions, we also fit participant and group-level psychometric curves as cumulative Gaussians using the Psignifit Toolbox for MATLAB (Wichmann & Hill, 2001; Fründ et al., 2011) and fit chronometric data with a scaled logistic function using a nonlinear least squares method. From the psychometric fits, we extracted the 75% points discrimination threshold (thr) and computed the discrimination slope (slo) based on the difference threshold, which is different from thr and a function of the slope and the midpoint percentile for 2AFC tasks π (= 75%), described by (Equations 1) and (2):

(1) $$dif\;ference\;threshold\;=\;\frac{1}{slope}*log\frac{\pi }{1-\pi }$$

(2) discriminationslope=2*differencethreshold

### Reference frame analyses

We then performed a reference frame analysis on the observed behavioral effects for each rotation condition. To do this, we first made predictions for these effect sizes proportional to the complexity of the RFT in each reference frame, then computed *R*-squared coefficients for changes (relative to the nonrotated condition) in RT, percent correct, and reward rate. Transformation complexity is defined by the angular rotation required between the assumed visually encoded evidence reference frame and the response reference frame, which has different relative angles depending on motor effector (saccade or button, which are head- or shoulder-centered, respectively). For example, in the H-nS condition with rightward motion and a 30° head roll (and 10% ocular counter roll of 3°), we may assume an eye-centered input reference frame and a shoulder-centered, button press response; for a spatially horizontal (0°) response, this results in a required rotation of *RFT* *rotation* = *abs*([*response* *rotation*] − [*encoded* *rotation*] ) or *abs*([0°] − [(0° − (30° − 3°)]) = 27°, representing a qualitatively large expected effect size. In the H-S condition with 45° motion and 30° head roll and an assumed head-centered encoding of motion evidence, a spatially horizontal, 0° saccade response requires an *abs*(30° − 15°) = 15°  rotation, or a qualitatively intermediate expected effect size. In our reference frame analysis of the effects, we used these rotational requirements to derive general predictions for each reference frame, with coarse effect size expectations (see Figure 6A for visual representation across conditions). Briefly, null, intermediate, and large effect sizes were represented by 0, 0.5, and 1, respectively. Together, these effect size predictions provided a theoretical framework within which we could quantify the effects relative to different coordinate systems.

### Statistical analyses

We performed several *n*-way analyses of variance (ANOVAs) (with four factors plus interaction terms) to account for variance in decision-making behavior (across RT, percent error, and reward rate) due to coherence level, RFT requirements, participant, and motor effector. To correct for statistical sampling error, we also carried out a multiple-comparison procedure based on Tukey's honestly significant difference criterion. We used the 95% confidence intervals estimated using Monte-Carlo simulations (Wichmann & Hill, 2001; Fründ et al., 2011) to compare 75% discrimination thresholds and slopes across RFT conditions in our psychometric analyses. For group-level, cross-condition comparisons, we used paired *t* tests.

## Results

We utilized several different rotational conditions to determine how the misalignment between stimulus and response reference frames affected decision-making. Different response modalities required different RFTs when stimulus and/or head rotation was imposed for saccade-related (eye-to-head) and button press–related (eye-to-shoulder) performance of a 2AFC perceptual decision task. Using these conditions, we analyzed the effects of RFTs on speed (RT), accuracy (percent error), and net performance (reward rate). This approach allowed us to determine both if changing the RFT requirements had any effect on the integration of decision evidence and, if so, if these effects revealed anything about the coordinate frames of the neural circuitry underlying these decisions.

### Head and stimulus rotations induced distinct effects on response times and task performance across conditions

We measured the 3D head (i.e., yaw, pitch, and roll) and two-dimensional eye orientation (i.e., horizontal and vertical) while participants performed the task. Importantly, the degree to which head roll and ocular torsion (assuming a small, ∼10% contribution from head roll–induced counter-roll) must be accounted for in each condition's visual-to-motor transformation defined the RFT complexity between visual and motor coordinates in our task (see Materials and methods for details). At the group level, participants were consistent in head roll magnitude from condition to condition. For the four head rolled conditions across each effector, participants rolled their heads on average between 28° (minimum) and 32° (maximum), with standard deviations between 7° (minimum) and 9° (maximum) across all participants.

**Figure 3.**
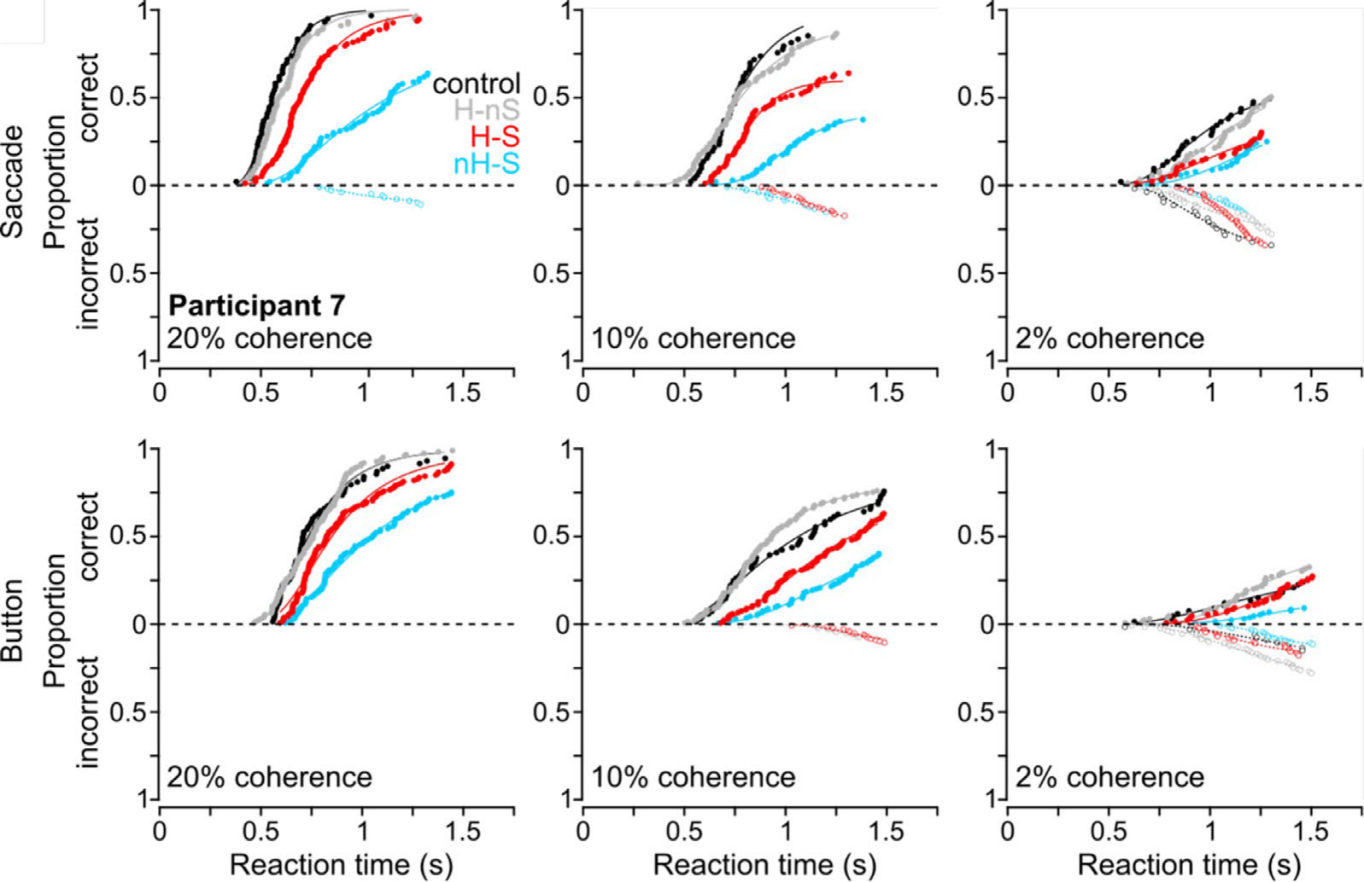
Single participant cumulative RT distributions. Across coherence levels (columns), specific patterns in RTs across rotational conditions (color-coded, see legend) are shown for Participant 7. Differences in the order of these RT distributions can be seen when comparing saccade (top row) to button responses (bottom row).

We found that head and stimulus rotations induced different effects on RT and accuracy depending on condition. As shown in Figure 3A (e.g., Participant 7), cumulative distributions of RTs showed that, depending on the rotation condition, the estimated median RTs shifted by various amounts relative to the control condition in which the head was upright and the stimulus motion axis was horizontally oriented. We also observed overall increases in RT and decreases in accuracy with task difficulty (20% to 10% to 2% motion coherence), with each condition inducing different effect magnitudes. These effects depended on the response type, suggesting a systematic role for the transformation required to convert sensory input into the response frame used for decision-making.

### RT and percent correct varied with effector, but there was no speed–accuracy trade-off

**Figure 4.**
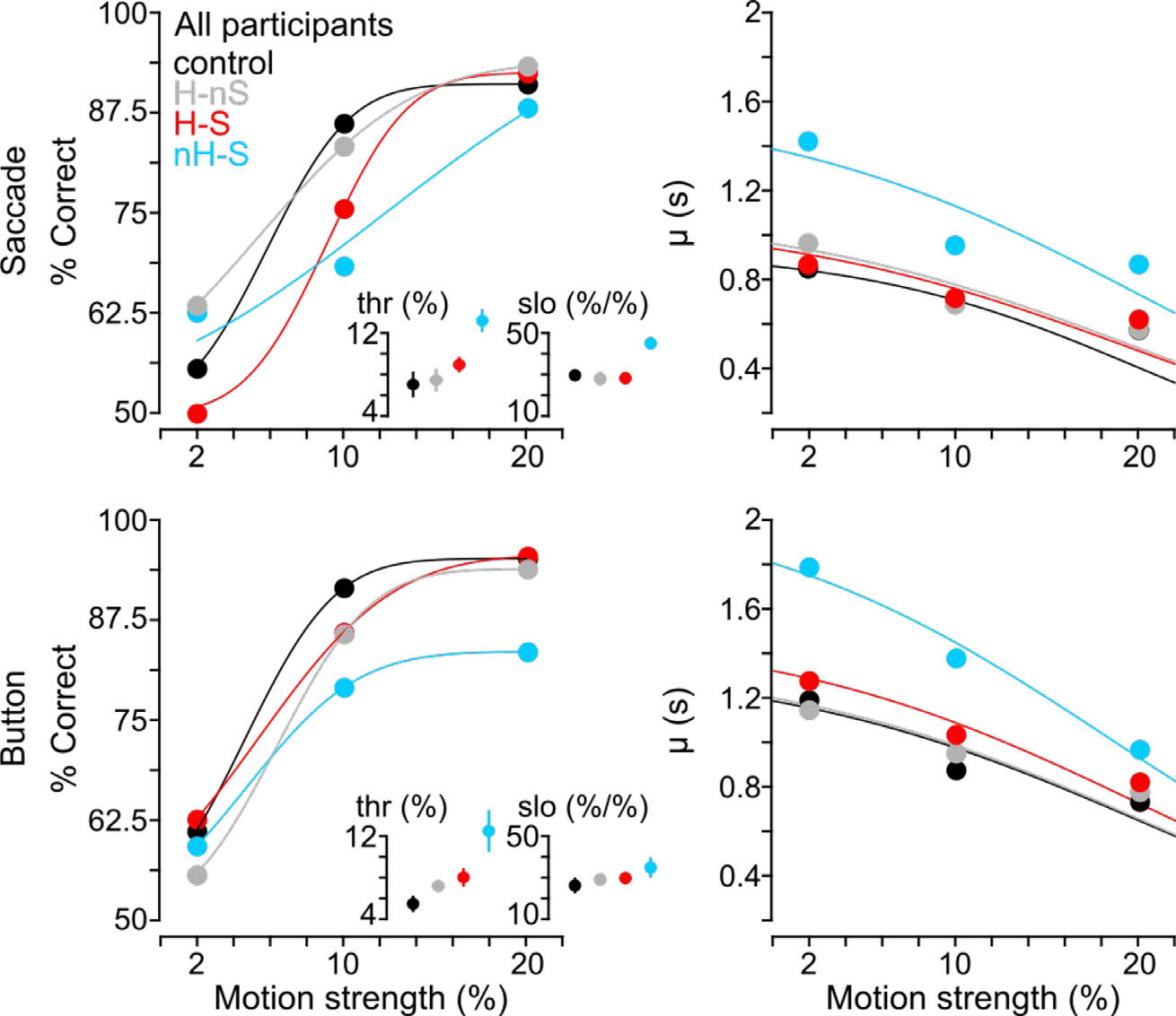
Psychometric and chronometric functions. Group-level psychometric and chronometric functions revealed that speed and accuracy were not traded off across rotation conditions, as participants were generally less accurate (psychometric functions, left column) and also slower (chronometric functions, right column) under rotated conditions. In the chronometric plots, each point represents the group average of the LATER fit parameter µ approximating the median reaction time of each condition at each motion strength. Left insets show the discrimination thresholds (thr), which represent the threshold coherence (%) at which participants chose the correct direction 75% of the time for the 2AFC task. Right insets also show the discrimination slope (slo), which approximates the sensitivity to motion strength.

We analyzed RT and accuracy effects relative to control across response type and rotation condition. Figure 4 illustrates this phenomenon with psychometric and chronometric functions at the group level. Psychometric functions (left column) show that behavior qualitatively differed between conditions depending on whether participants responded with a saccade or button press and that the nH-S condition (cyan) in general saw the worst task performance. Note that these fits were meant only to compactly describe the data points within the motion stimulus range tested and allowed us to coarsely examine any relative speed accuracy trade-offs for the conditions; these fits are not meant to be interpreted as one would interpret classic psychometric functions. A repeated-measures ANOVA detected a main effect of rotation condition (*F*(3) = 2.87, *p* < 0.05) and an interaction effect of response type and rotation condition on the discrimination threshold (thr) and slope (slo) (*F*(3) = 7.73, *p* < 0.01), although we did not find a main effect of response type (*p* = 0.08). A one-way ANOVA detected main effects of motion coherence (*F*(2) = 20.04, *p* < 0.01), response type (*F*(1) = 11.67, *p* < 0.01), and rotation condition (*F*(3) = 10.77, *p* < 0.01) on reaction time (µ). Taken together, these observations suggest that there was an overall degradation of the encoded evidence but no clear speed–accuracy trade-off across rotational conditions (Standage, Wang, et al., 2014). Additionally, the observed response type–specific patterns of performance changes suggest that the reference frame of the motor response played a role in the encoding of evidence.

**Figure 5.**
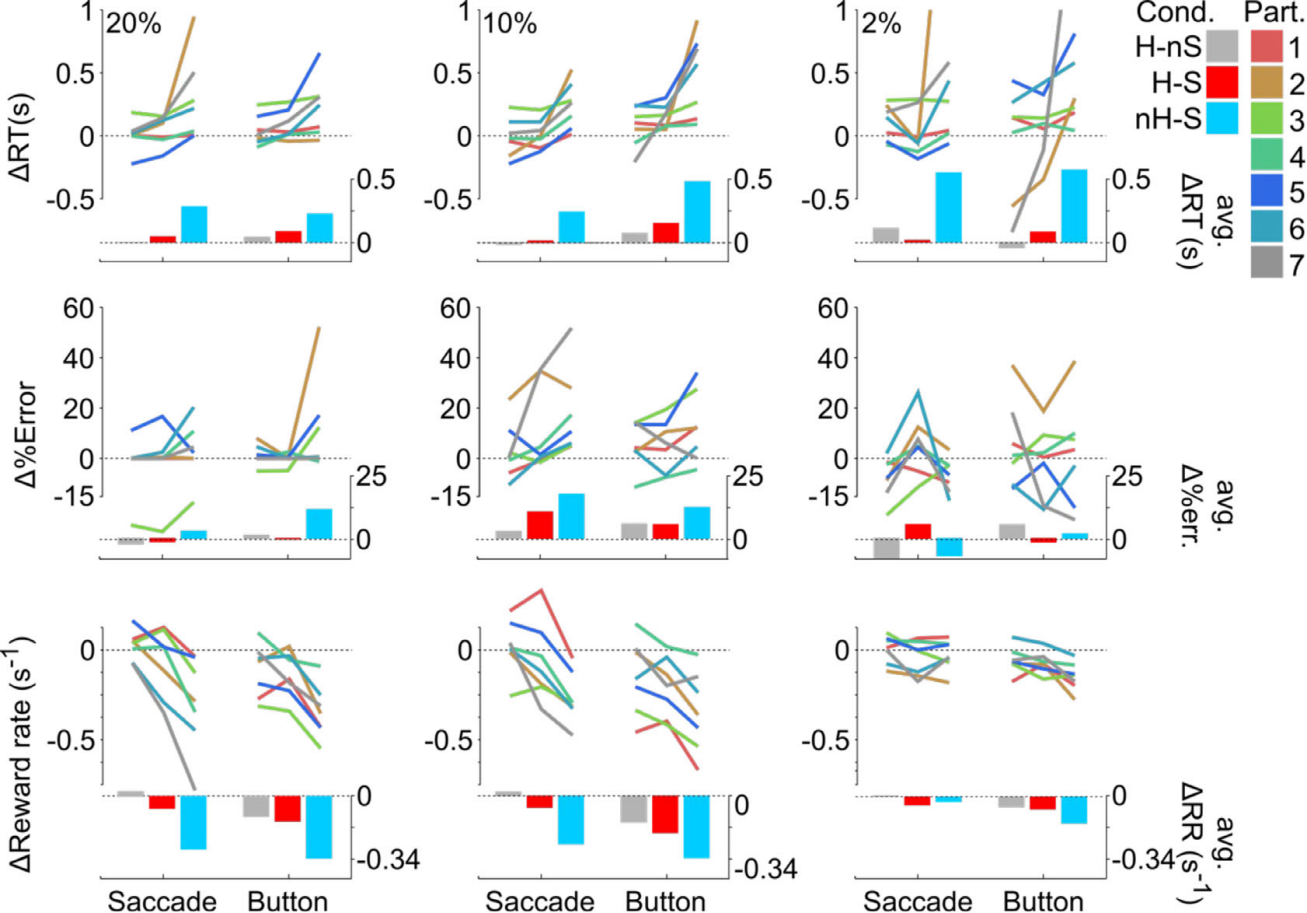
Variability of rotational effects on performance across participants. Changes in reaction time (top row), percent error (middle row), and reward rate (bottom row) across coherence level (columns), with left axes representing scale for single participant changes (colored line segments, see legend for participant numbers) and right axes representing group-level average changes across rotation conditions (color-coded bars). Each vertex of the line segments represents one rotation condition, in line with the colored bars at the bottom.

To better understand these results, we analyzed behavioral task performance compared to the baseline control condition across experimental conditions and participants in Figure 5 through several ANOVAs. We observed trends consistent with a degradation of evidence encoding such that the task was more difficult under rotated conditions. Across task difficulty, we found that RT increased (*F*(2) = 12.73, *p* < 0.01), percent error increased (*F*(2) = 326.5, *p* < 0.01), and reward rate decreased (*F*(2) = 33.54, *p* < 0.01). We also found a significant main effect of rotation condition on RT (*F*(3) = 7.78, *p* < 0.01), percent error (*F*(3) = 4.76, *p* < 0.05), and reward rate (*F*(2) = 34.25, *p* < 0.01). We found that response type only affected reward rate (*F*(1) = 21.58, *p* < 0.01). On average (inset bars on right axes), participants had longer RTs and had lower reward rates when making decisions under the nH-S condition (cyan bars), when compared to control (Tukey's honestly significant difference procedure multiple comparison *p* < 0.05), H-nS (gray; multiple comparison *p* < 0.05), and H-S (red; multiple comparison *p* < 0.05) conditions. Importantly, we did not see a speed–accuracy trade-off (e.g., faster/slower responses and higher/lower percent error), as reward rate also decreased (bottom row) with increases in both RT and percent error. We observed participant-specific differences in RT between response types (interaction effect, *F*(6) = 4.93, *p* < 0.01) and between RFT condition (interaction effect, *F*(18) = 3.03, *p* < 0.01). For example, one can see differences between saccade and button responses for Participant 5 or for Participant 3 across each response type and coherence level (see Figure 5). This trend suggests that the noise added to the evidence encoding not only changed with response type but also with rotational condition, in agreement with the observed changes to psychometric and chronometric functions. We next used a reference frame approach to determine the source of this added noise in the decision process.

### Reference frame analysis

To quantify this interparticipant variability, we interpreted the effects using predictions from stochastic reference frame transformations from Alikhanian et al. (2015). Head roll and ocular torsion angles are represented as noisy sensorimotor estimates in the brain. Alikhanian et al. (2015) showed that using noisy angles to perform an angular coordinate transformation (such as in our rotation paradigm) is expected to add noise to the transformed retinal inputs, leading to trial-to-trial variability in the decision process. Here, we quantify the effects of this added noise based on different RFT rotation requirements for different sensorimotor coordinates (eye, head, and shoulder centered). Briefly, for saccade and button responses (head or shoulder centered, respectively), we computed the expected sensory-to-motor transformation rotation angle between different sensory coordinate frames. We assumed that the motion information used in the decision was impaired to an extent that was proportional to the overall required visuomotor rotation.

**Figure 6.**
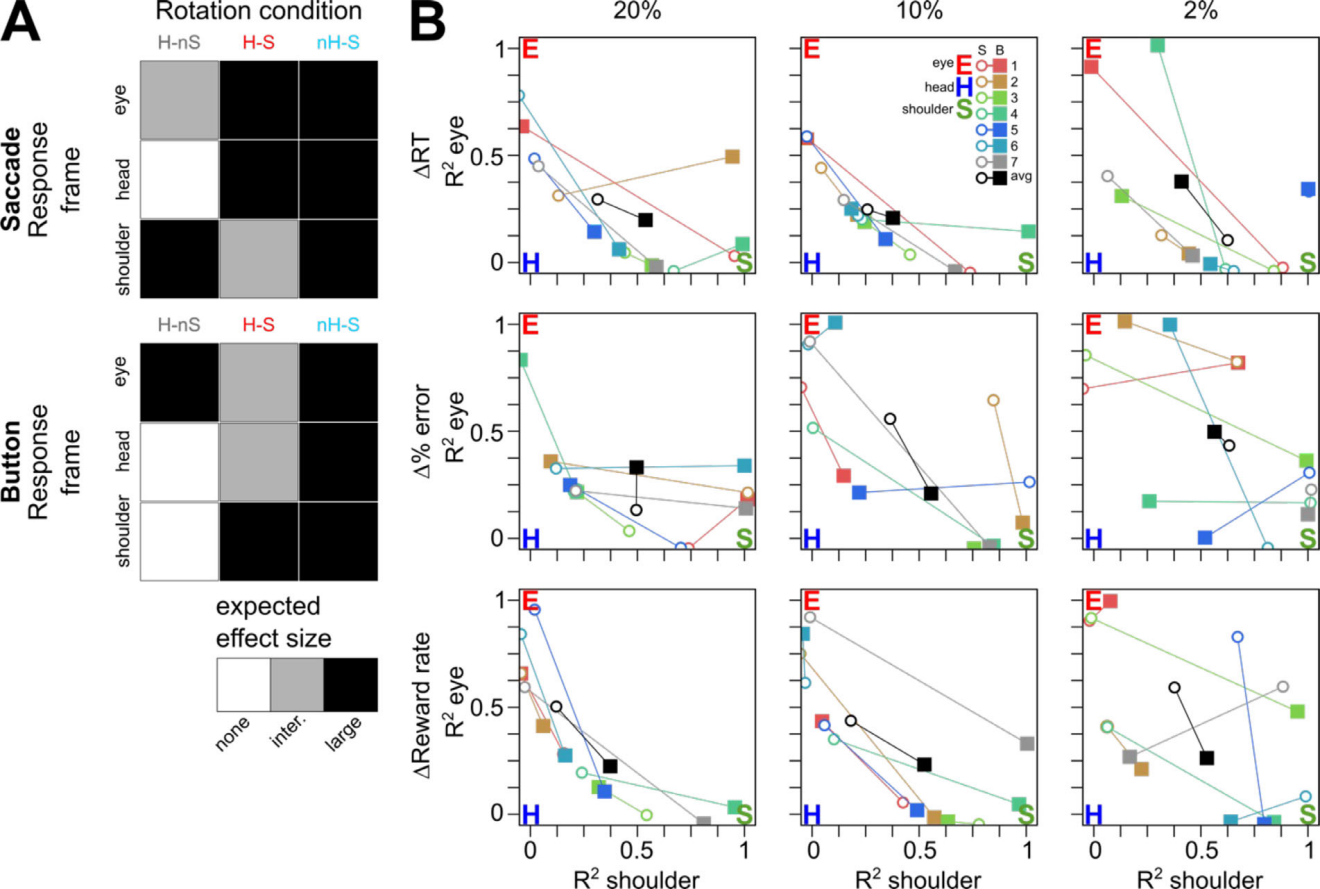
Reference frame predictions and analysis. (**A**) Response type–specific reference frame prediction matrices. Each cell represents a specific reference frame and the predicted effect size for the corresponding rotation condition. For example, if motion evidence were coded according to an eye-centered reference frame, for the condition in which only the motion stimulus were rotated (condition nH-S), we would expect a large (black shading) reference frame transformation-induced stochastic effect on the coded evidence signal in both saccade and button response conditions. (**B**) Participant *R*-squared coefficients for correlation analysis between prediction matrices in panel (A) and observed changes in reaction time (top row), percent error (middle row), and reward rate (bottom row), across coherence levels (columns). Participant color code is the same as in previous figures, and black symbols represent across-participant means. Open circles and filled squares represent *R*-squared coefficients for saccade responses and for button responses, respectively. Pure eye-centered (red), head-centered (blue), and shoulder-centered (green) reference frame predictions are represented with large filled circles. Note that we have plotted the eye–shoulder projection of this 3D space (thus the head *R*^2^ axis is along the origin).

This provided predictions for the size of each effect, relative to the head-upright, motion horizontal control, according to the required rotation for a correct motor effector–centered response in each condition, which we illustrate in Figure 6A. For example, consider the eye-centered prediction for the condition in which both the head and the screen were rotated and a saccadic response was required (H-S; middle cell, top row, top grid, Figure 6A): In order to correctly interpret the spatial motion direction using eye-centered information, the brain must rotate the retinal vector (which points along its horizontal axis; for visualization, see Figure 2A) by the head roll magnitude to generate a screen-centered horizontal saccade. This requirement differs for the condition in which the head, but not the stimulus, was rotated (H-nS). Because the retinal vector was rotated solely by head roll and ocular counter-roll, and the eyes were also rotated along with the head, the brain only needed to account for ocular counter-roll when transforming the retinal vector into a screen-horizontal saccade. Therefore, in the eye-centered case, we predicted a large stochastic effect for H-S (Figure 6A, black shading) due to head roll and an intermediate effect for H-nS (Figure 6A, gray shading) due to only ocular counter-roll. In our correlational analysis, we assume that a large effect = 1, an intermediate effect = 0.5, and a null effect = 0. In this way, we made predictions for each response type and for each reference frame (eye, head, and shoulder).

Using these predictions, we computed the *R*-squared coefficients for each behavioral parameter (RT, percent error, and reward rate), each participant, each response type, and each motion coherence. These are depicted in Figure 6B along with the predictions for purely eye-centered (red E), head-centered (blue H), and shoulder-centered (green S) codings. Each *R*-squared coefficient is color-coded according to participant and represented by a symbol depending on response type (saccades: open disk; button: filled square). Across both RT and percent error at 20% coherence, the *R*-squared coefficients suggest that evidence was being encoded according to a continuum of reference frames between eye and shoulder, with a strong head-centered component in some cases (e.g., button press responses of Participant 5).

The transformation-related effect was also dependent on the strength of the stimulus, indicating that the addition of variability to the encoded evidence depended on the initial strength of visual motion. For example, while there is a clear organization of *R*-squared coefficients for the 20% and 10% motion coherence conditions for changes in reaction time along an eye–head–shoulder continuum (Figure 6B, upper left and middle panels), this continuum becomes less clear when the stimulus strength is decreased at 2% motion coherence (Figure 6B, upper right panel).

With this analysis, we quantified the response type–specific component that we initially observed in the psychometric and chronometric functions (Figure 4). This component was strongest when considering reward rate (bottom row of Figure 6B). Across motion coherence, group reward rate averages (black symbols) indicated that evidence leading to saccadic responses was more eye centered while evidence leading to button responses was more shoulder centered. This trend suggests that the neural circuitry encoding decision evidence is tied to the motor plan for the upcoming movement. Additionally, this mixture of eye- and shoulder-centered components indicates that there could be some concomitant evidence coding by eye- and shoulder-related areas during integration, regardless of eventual motor effector.

### Evidence for stochastic facilitation

The idea of stochastic facilitation is that increases in variance (noise) of the sensory decision signals in the brain can alter the decision dynamics (Standage et al. 2014) to potentially produce faster decisions. These new dynamics may result in a speed–accuracy trade-off (e.g., with no net effect on reward rate) or accuracy may stay the same despite the faster decision (facilitating an increased reward rate). The increase in variance during head roll is posited to arise from signal-dependent noise in the estimated head angle. Therefore, we paired conditions with identical (as much as possible) sensory and motor reference frames. Even for between-condition comparisons in which there was no change in the overall visuomotor rotation for the RFT, the transformation still relies on noisier estimates for head roll when the head is eccentric than when the head is upright. Thus, if increases in head roll noise lead to increases in stochasticity of RFTs, this could produce stochastic facilitation in decision-making.

**Figure 7.**
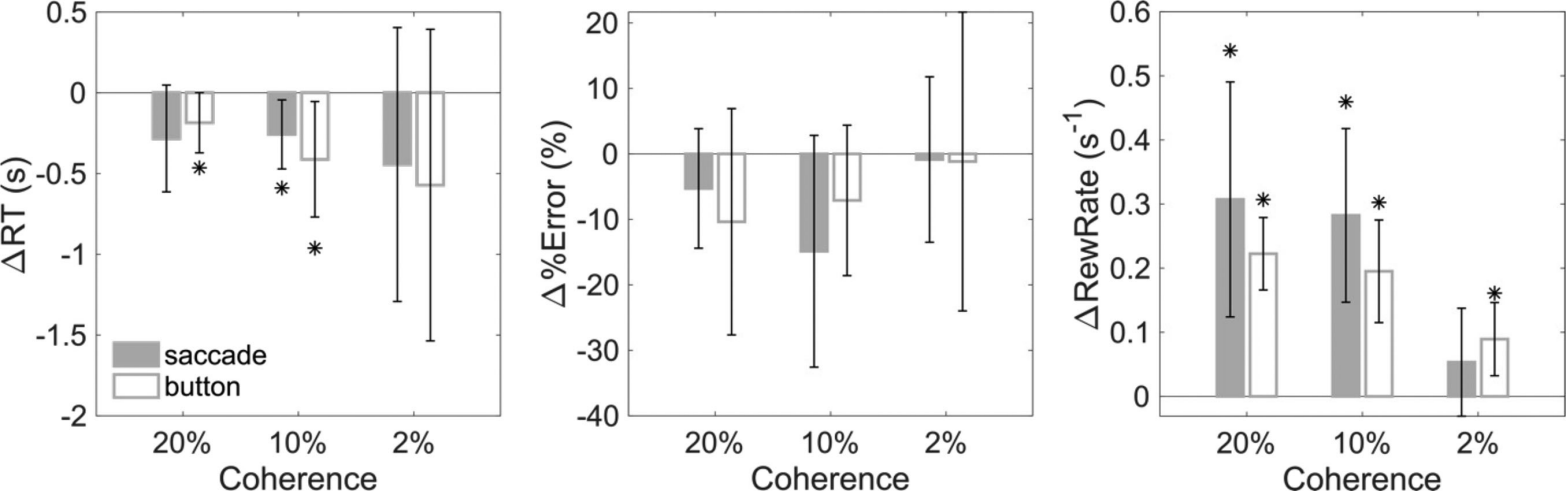
Stochastic facilitation for decisions under H-nS conditions versus nH-S conditions. Delta reaction times (left), percent errors (middle), and reward rates (right) for H-nS for saccades (filled bars) and button presses (open bars). Asterisks represent significant differences from nH-S conditions using a paired *t* test.

To discern any effects of head roll on the decision process, we paired the H-nS condition with the nH-S condition for both button press and saccadic responses and compared the reaction time, percent error, and reward rates. Importantly, for these conditions, the retino-spatial rotation is similar (with the difference arising from head–screen axis misalignments, plus ocular counter roll), but in one case, the head is rolled (H-nS), while in the other, it is not (nH-S). The results of this analysis are shown in Figure 7 for button press (open bars) and saccadic (solid bars) responses. We observed a significant increase in reward rate for all but the 2% coherence-level saccadic response conditions, in agreement with the stochastic facilitation hypothesis (paired *t* tests, all *p* < 0.05). This increase in reward rate was largely driven by a decrease in reaction time (paired *t* tests, *p* < 0.05 for 20% button press and 10% both response types; no significant difference for percent error), suggesting faster decision dynamics under head-rolled conditions. Note that we did not compare the control condition with the H-S condition due to the presence of oblique retinal motion in this condition (due to ∼30° head tilt and 45° on screen motion tilt), which created an unfair comparison with the horizontal control motion vector due to the oblique effect (Appelle, 1972).

## Discussion

### Summary of findings

The goal of this study was to determine the influence of stochasticity from visuomotor transformations on perceptual decision-making in a 2AFC visual motion discrimination task. We designed a paradigm in which seven participants performed the task under several rotation conditions in which the head and/or stimulus were rotated. We behaviorally quantified RFT-based speed and accuracy effects, including any changes indicative of dynamic stochastic facilitation. We found that (1) in general, stochastic reference frame transformations impair decision-making, leading to slower, less accurate decisions; (2) this stochasticity is added in a manner consistent with a mixed eye–head–shoulder representation of evidence; and (3) within this continuum, there is an effector-specific component, with saccadic responses more closely resembling eye-centered predictions and button responses more closely resembling shoulder-centered predictions. Furthermore, we also found evidence for stochastic facilitation when we compared conditions in which the net retino-spatial mismatch was identical, but the head was rolled in one condition. This observed facilitation effect was dependent on both the signal-to-noise ratio of the sensory evidence (i.e., coherence) and the magnitude of visuomotor rotation. Our findings are consistent with the hypothesis that perceptual decision-making and visuomotor reference frame transformations occur within the same neural circuitry (Dorris et al., 1997; Gold & Shadlen, 2000) and as such are consistent with the affordance competition hypothesis of embodied decision-making, which predicts that motor planning for perceptual decision-making occurs in parallel between networks coding for multiple potential actions (for reviews, see Cisek 2007; Cisek & Pastor-Bernier, 2014).

Although both evidence integration and motor preparation are often necessary for choice behavior, it is often difficult to distinguish between the contributions of each using standard perceptual tasks. Previous efforts to do so include using delays between stimulus viewing and motor response (Shadlen & Newsome, 2001; Sommer & Wurtz, 2001; Lemus et al., 2007), limiting stimulus viewing time (Bergen & Julesz, 1983; Ratcliff & Rouder, 2000; Bodelón et al., 2007; Kiani et al., 2008) and even “compelling” the movement by informing the perceptual system ahead of time about the target characteristics (Salinas et al., 2014). At the neural level, perceptual and motor processes both occur in sensorimotor association areas (Munoz & Wurtz, 1995; Dorris et al., 1997; Horwitz & Newsome, 1999; Shadlen & Newsome, 2001; Hernández et al., 2010; Costello et al., 2013; Mante et al., 2013). Not only are our findings consistent with these neurophysiological principles, but we have also now quantified this inseparability for the first time within an RFT framework.

Decisions in our experiment were not always impaired by head roll, however. We observed stochastic facilitation of decisions during head-rolled conditions with a large visuomotor rotation compared to those without head roll (i.e., H-nS compared to nH-S). This finding suggests that a more variable estimate of head roll angle for eccentric head orientations modulates decision dynamics without further degrading the motion evidence for already large visuomotor rotations. World motion direction also played a significant role in the perceptual decision process; indeed, world-horizontal conditions (control, H-nS) always led to faster decisions than world-oblique conditions (nH-S, H-S). However, oblique motion alone cannot explain the difference in response times between nH-S, H-S, and H-nS we observed, and this oblique effect cannot be disentangled from the RFT due to the need to generate a world-horizontal eye movement/button press in all conditions. Thus, we believe both the RFT and world-motion direction had an influence on the decision process. Noise from RFTs appears to affect perceptual decisions in a way that depends on the full visuomotor context.

### Open questions

We found that all the rotation conditions we applied impaired decision-making relative to nonrotated control conditions. The corresponding systematic changes in LATER model fit parameters suggested that this effect is a direct result of a degradation of the encoded visual motion signal. In the neural circuitry, this effect would most likely occur in the middle temporal (MT) or medial superior temporal (MST) areas (Albright, 1984; Britten et al., 1992, 1993, 1996; Salzman et al., 1992; Inaba et al., 2007). MT and MST are highly interconnected areas that serve as the interface between retinal motion signals and the rest of the visuomotor pathways (Ungerleider & Desimone, 1986; Komatsu & Wurtz, 1988; Newsome et al., 1988; Ilg & Their, 2003; Inaba et al., 2011; for review, see Krauzlis, 2004) and exhibit gain modulation and receptive field shifts (Chukoskie & Movshon, 2009; Fujiwara et al., 2011; Inaba et al., 2011) mechanistically consistent with carrying out 3D visuomotor transformations (Blohm & Crawford, 2007; Blohm et al., 2009; Blohm & Lefèvre, 2010; Blohm, 2012; Murdison et al., 2015). If these areas indeed provide the neural substrate for the addition of variability to visual motion signals via RFTs, then gain modulation for RFTs itself could be a stochastic process—a possibility that could be investigated in future electrophysiological and modeling work.

The finding that group-level behavioral effects could be captured by a continuum of eye-, head-, and shoulder-centered signals suggests that the underlying encoded decision evidence should be at least partially shared between motor effectors. The considerable interparticipant variability of this effect, however, remains unexplained. For each participant, RFT stochasticity added to the integration of evidence could result in a unique level of reliability for the population “readout” of the current decision signal by downstream neural areas, resulting in a certain amount of variability in RT distributions (Carpenter & Williams, 1995). Possibly due to idiosyncratic differences in adeptness at the visuomotor task (e.g., some participants may have had better eye–hand coordination than others), we would expect each participant to exhibit different levels of vulnerability to RFT stochasticity. Differences in how population output responses are decoded by structures closer to the motor output such as the superior colliculus (SC) (Munoz & Wurtz, 1995; Dorris et al., 1997; Horwitz & Newsome, 1999; Sommer & Wurtz, 2001) or primary motor cortex (M1) (Riehle & Requin, 1989; Crammond & Kalaska, 1996, 2000) could potentially explain some of the interparticipant variability we observed in RT, percent error, and reward rate correlations.

### Potential mechanism and underlying neural circuitry

Our findings are consistent with the hypothesis that the encoding of motion evidence is degraded by RFTs; however, this effect is not the only possible way that RFTs could affect decision-making. For example, changes in background noise could have modulated the dynamics of circuitry integrating evidence (Furman & Wang, 2008; Roxin & Ledberg, 2008; Standage et al., 2013; Standage, Wang, et al., 2014; for review, see Standage, Blohm, et al., 2014). If so, a speed–accuracy trade-off would likely have been observed.

The finding that the impairment of performance relied partially on the response type implies the existence of two partially distinct perceptual decision-making networks between behavioral responses, as previously theorized (Dean et al., 2011; Madlon-Kay et al., 2013). In the macaque lateral intraparietal area (LIP) and the parietal reach region (PRR), which lies along the medial bank of the intraparietal sulcus (IPS), population-level neural activity has been shown to reflect an effector-nonspecific movement signal until a monkey makes a decision regarding which motor effector to use, at which point PRR activity is associated with a reach (Cui & Andersen, 2007; Yttri et al., 2014; Wong et al., 2016) or LIP activity is associated with a saccade (Cui & Andersen, 2007; Wong et al., 2016). In this regard, recent electrophysiological findings (Wong et al., 2016) indicate that there are ensembles of neurons on both the medial and lateral banks of the IPS that are active during the decision process. Specifically, Wong and colleagues (2016) found an ensemble of neurons that predict the upcoming decision, independent of effector-specific region, that coherently spike prior to effector-specific local ensembles in each bank, consistent with previous findings (Cui & Andersen, 2007; Yttri et al., 2014). These partially distinct neural ensembles could therefore give rise to the mixture of reference frames our perceptual findings imply should be present in the neural integration of motion evidence. Of course, this explanation does not preclude perceptual and motor contributions from other effector-nonspecific areas such as the prefrontal cortex (Madlon-Kay et al., 2013) or from other effector-specific areas whose activities are believed to implement a decision variable such as FEF (Hanes & Schall, 1996; Gold & Shadlen, 2000, 2003; Sommer & Wurtz, 2001) or the dorsal premotor cortex (Crammond & Kalaska, 1996, 2000, Cisek & Kalaska, 2002, 2005), or downstream (or possibly via bidirectional projections) in SC (Munoz & Wurtz, 1995; Dorris et al., 1997; Sommer & Wurtz, 2001; White et al., 2013) or M1 (Riehle & Requin, 1989; Crammond & Kalaska, 1996, 2000). The precise role that RFT stochasticity plays within such a distributed perceptual decision network, especially with several anatomically distinct sensorimotor association areas with different physiological properties and latencies, is unclear (Siegel et al., 2015). Furthermore, within these areas, it is also unclear how local neural population codes vary with body and spatial geometry during visuomotor decisions. These are questions that could be further investigated psychophysically and electrophysiologically.

Stochasticity is a hallmark of neural systems (Faisal et al., 2008; McDonnell & Ward, 2011) and can have benefits for neural processing; for example, noise can improve signal detection because it brings subthreshold membrane potentials closer to firing threshold (Aihara et al., 2010; McDonnell & Ward, 2011). This stochastic facilitation can have behavioral benefits (Uhlhaas & Singer, 2006), such as from improved contrast detection (Collins et al., 1996; Wells et al., 2005; Funke et al., 2007; Starzynski & Engbert, 2009), improved speech perception (Kishon-Rabin et al., 2008), or preventing deadlock in decision-making (Deco et al., 2009). Most previous studies have focused on how stochastic noise affects information *coding*. Here we show that it can also benefit information *processing*, as predicted by Standage, Wang, et al. (2014). Based on this theoretical study, we hypothesize that increasing noise levels will increase the responsiveness of the competing neural populations—in areas presumably including FEF—accelerating the competition and leading to faster decisions. Our findings are in line with studies reporting cross-modal noise benefits for signal processing (Lobel et al., 1998; Usher & Feingold, 2000; Freedland et al., 2002; Willems et al., 2007; Smith et al., 2010; Wilkinson et al., 2010; Kaut et al., 2011) showing that stochastic facilitation can enhance seemingly unrelated neural computations (e.g., induced vestibular noise enhances memory). Thus, supposedly undesirable noise can have very positive effects for the brain and behavior. Indeed, from a Bayesian perspective, without noise, it would be impossible for the brain to adapt, learn, and integrate sensory signals (Deneve et al., 2001; Todorov & Jordan, 2002; Beck et al., 2008; Ma et al., 2008).

Our findings have implications for studies involving the integration of visual evidence for movement, whether used for perceptual decision-making or motor preparation. First, we found that RFT stochasticity affects the encoding of evidence for perceptual decision-making, bringing to light the requirement for controlling the visuomotor geometry during perceptual tasks. Second, the finding that this phenomenon added variability was partially effector specific could explain some variability between psychophysical performance when the perceptual task is identical, except for the motor response (Palmer et al., 2005).

The influence of RFT stochasticity on perceptual decision-making is consistent with previous findings in visuomotor tasks (Sober & Sabes, 2003, 2005; Schlicht & Schrater, 2007; McGuire & Sabes, 2009; Burns & Blohm, 2010; Burns et al., 2011), suggesting that it represents a generalized phenomenon wherever RFTs can be found throughout the perceptual and motor systems. Whether this phenomenon can be further extended to processes requiring a higher degree of cognitive involvement such as strategic decision-making or memory storage and retrieval remains an open question.

We noted in the introduction section that animals typically keep their head upright, even if this was not energetically beneficial. Here we showed that tilting the head can have a beneficial effect regarding the speed and accuracy of perceptual decision-making. While keeping the head upright with respect to gravity might thus be suboptimal, there are many other considerations for why this might be the best strategy. First, as mentioned in the introduction section, an upright head is believed to minimize vertical disparity. Furthermore, in the wild, a tilted body with an upright head means that the head-on body orientation is actually rolled and the head typically experiences centrifugal forces leading to increased neck muscle contractions. Past research (Abedi Khoozani & Blohm, 2018) has shown that neck muscle contraction adds noise to RFTs in a multisensory reaching task. If this were also applicable here, then this added noise could actually lead to stochastic facilitation, while a spatially upright head would also minimize vertical disparity. Thus, our findings might be perfectly in line with optimal behavior in the wild.
